# Decoding the Effects of High Hydrostatic Pressure and High-Temperature Short-Time Sterilization on the Volatile Aroma Profile of Red Raspberry Juice

**DOI:** 10.3390/foods13101574

**Published:** 2024-05-18

**Authors:** Wentao Zhang, Xuejie Li, Xuzeng Wang, He Li, Xiaojun Liao, Fei Lao, Jihong Wu, Jian Li

**Affiliations:** 1 School of Food and Health, Beijing Technology and Business University, Beijing 100048, Chinaxuzeng@st.btbu.edu.cn (X.W.); lihe@btbu.edu.cn (H.L.); 2 College of Food Science and Nutritional Engineering, China Agricultural University, Beijing 100083, China; liaoxjun@cau.edu.cn (X.L.); fei.lao@cau.edu.cn (F.L.); 3 Key Laboratory of Green and Low-Carbon Processing Technology for Plant-Based Food of China National Light Industry Council, Beijing 100048, China; 4 Beijing Engineering and Technology Research Center of Food Additives, Beijing 100048, China

**Keywords:** red raspberry juice, C6 aldehyde aroma-active compounds, high-hydrostatic pressure sterilization, high-temperature short-time sterilization, fatty acid metabolism

## Abstract

The loss of distinctive aromas due to sterilization significantly hinders efforts to enhance the sensory quality of fruit and vegetable juices. This study aimed to elucidate the impacts of high-hydrostatic pressure (HHP) and high-temperature short-time (HTST) sterilization methods on the loss of C6 aldehyde aroma-active compounds in red raspberry juice. External standard quantification and quantitative descriptive analysis (QDA) revealed a notable decline in the levels of hexanal and (*Z*)-3-hexenal following the HHP and HTST treatments (*p* < 0.05), resulting in a marked attenuation of the grassy aroma characteristic of red raspberry juice. Furthermore, a comprehensive examination of the precursors, pivotal enzymes, intermediates, and downstream aromas within the fatty acid metabolism pathway in different raspberry juice samples indicated that the C6 aldehydes loss induced by HHP and HTST sterilizations was primarily ascribed to the competitive inhibition of β-oxidation and the hindered enzymatic oxidation of fatty acids. These insights suggest that modifying sterilization protocols and enhancing enzymatic stability may help preserve the aroma integrity of raspberry juice. Our findings offer practical guidance for optimizing juice processing techniques to maintain flavor.

## 1. Introduction

Red raspberries (*Rubus idaeus* L. cv. Heritage) are favored by consumers for their plump and juicy fruit, rich and vibrant color, and strong, distinctive flavor. Additionally, they are abundant in bioactive components, such as raspberry ketone (RK), ellagic acid, and superoxide dismutase (SOD), which have been demonstrated to effectively reduce the prevalence of chronic diseases, including cardiovascular disease [1], type II diabetes [2], and lung cancer [3] with long-term consumption. However, due to their high water content and short shelf life, fresh red raspberries often suffer significant losses during storage and transportation. To mitigate this, approximately 684,000 tons of fresh red raspberries are processed into various products annually to extend their shelf life [4]. Among these products, red raspberry juice has become one of the most popular in the market, as it best preserves the original flavor and nutrients of the fruit [5].

Flavor plays a critical role in the quality and consumer acceptability of fruit and vegetable products [6]. Research into the flavor components and their dynamic changes is invaluable for clearly understanding and controlling the key quality parameters that influence raspberry juice processing. To date, more than 200 types of flavor compounds, mainly including aldehydes, alcohols, ketones, and terpenes, have been identified in red raspberries and their processed products [7]. However, due to their odor thresholds, only a select few of these volatiles can be perceived [8]. In our previous study, 14 key aroma-active compounds with odor activity values (OAVs, representing the ratio of compounds concentration to their threshold) ≥ 1 were identified in red raspberry juice using a sensory-oriented flavor analysis method [9]. Among these volatiles, two C6 aldehydes, hexanal and (*Z*)-3-hexenal, were demonstrated to be essential for the grassy attribute of raspberry juice, as revealed by aroma recombination and deletion models.

As a typical heat-sensitive beverage, the quality of red raspberry juice, particularly its flavor, is significantly affected by processing treatments. Sterilization is an important unit in the production of red raspberry juice, which is crucial for ensuring food safety and extending the shelf life of products [10]. High-temperature short-term (HTST) sterilization is widely used in fruit and vegetable juice disinfection due to the advantages of strong practicability, easy operation, high efficiency, and low cost [11]. However, while killing microorganisms and inactivating enzymes, high temperatures may also have a negative effect on the sensory attributes and nutritional value of fruit and vegetable juices [12]. In recent years, nonthermal sterilization technologies involving high hydrostatic pressure (HHP) have better overcome the side effects of traditional thermal sterilization, such as slow heat transfer and large heat loss of nutrients, and have been increasingly used in the production of high-end fruit and vegetable products, such as nonconcentrated reduction juice [13].

Numerous studies have demonstrated that the sensory quality of fruit and vegetable juice is greatly influenced by various sterilization methods. For instance, Zhao et al. [14] compared the effects of HHP sterilization and thermal pasteurization combined with nisin on the quality of cucumber juice drinks, revealing that the levels of C9 odorants, such as (E)-2-nonenal and (*Z*)-6-nonenal, decreased significantly after HHP treatment. Similarly, Zhang et al. [15] investigated the effects of different sterilization techniques on the flavor profile of mango juice and observed that while HHP sterilization better preserved the original aroma profile compared to traditional thermal sterilization, it significantly altered substances linked to the grassy note, such as (E)-2-nonenal and (E, Z)-2,6-nonadienal. These findings suggest that while HHP offers advantages in maintaining overall flavor quality in fruit and vegetable juices, it may facilitate the loss of specific C6/C9 aldehyde aroma-active compounds, thereby affecting the perception of the grassy note.

Current research on sterilization methods for red raspberry juice has primarily focused on microbial inactivation, enzyme passivation, and anthocyanin degradation [16]. However, to the best of our knowledge, the impact of sterilization on the loss of key C6 aldehyde aroma compounds in red raspberry juice has not been extensively explored. In this regard, this study aims to (1) accurately quantify the contents of key C6 aldehyde compounds in HHP and HTST-treated red raspberry juice using an external standard method; (2) clarify the potential impact of aroma loss on the overall sensory quality of red raspberry juice by quantitative descriptive analysis (QDA); and (3) reveal the loss pathway of C6 aldehyde compounds by the non-targeted metabolome of fatty acids.

## 2. Materials and Methods

### 2.1. Plant Material

Red raspberry (*Rubus idaeus* L. cv. Heritage) fruits were cultivated and harvested from a local farm (41.533° N, 117.798° E, Chengde, Hebei province, China) in August 2018. The ripe fruits were picked, shipped, and selected as described in our previous work [9].

### 2.2. Preparation of Red Raspberry Juice

The collected red raspberries were rinsed with distilled water to remove loose dirt and grime from the surface. The drip-dried samples were squeezed to extract the juice using a household juicer (JYZ-E18, Joyoung Co., Ltd., Hangzhou, China). The mixture was centrifuged at 5000× *g* for 15 min at 4 °C (CR21GIII, Hitachi, Tokyo, Japan). Around 24.84 kg of fresh red raspberry juice was obtained from the supernatant filtered by cotton gauze. The resulting juice was manually filled into plastic bottles made from polyethylene terephthalate with a thickness of 0.065 cm. HHP and HTST treatments were conducted based on our previous work [17]. The HHP-treated juice was processed at 600 MPa for 10 min below 25 °C through an HHP unit (CQC30L-600, Suyuanzhongtian Scientific Co., Ltd., Beijing, China). Distilled water was used as pressure-transmitting fluid.

The pressurization rate was about 200 MPa/min, and the depressurization time was <3 s. The treatment time was the pressure-holding time, not including the pressure increase and release time. The HTST-treated juice was processed at 110 °C for 8.6 s through a pilot-scale pasteurizer with a tubular heat exchanger (FT74, Armfield Ltd., Hampshire, UK) and then cooled to 20 °C. The flow rate was 17.4 L/h. The rising time and cooling time were 320.3 s and 324.1 s, respectively. The prepared juices were immediately utilized for enzyme activity determination without undergoing freezing treatment. For other measurements, the juices were instantly frozen in liquid nitrogen and stored at −80 °C (DW-86L490, Haier Group, Qingdao, Shandong, China) for no more than two weeks.

### 2.3. Determination of the Volatile Compounds from Red Raspberry Juice

#### 2.3.1. Extraction of the Volatiles by HS-SPME

A multi-purpose sampler (MPS 2XL, Gerstel GmbH & Co. KG, Mülheim, Germany) equipped with the 50/30 μm divinylbenzene/Carboxen™/polydimethylsiloxane (DVB/CAR/PDMS) solid-phase microextraction (SPME) fiber (Supelco Inc., Bellefonte, PA, USA), was employed to extract the volatiles from the headspace of red raspberry juice [9]. For each sample, 7 mL of the clear accurately pipetted into a 20 mL headspace vial (ANPEL Laboratory Technologies Inc., Shanghai, China), followed by addition of 2.52 g of NaCl (Sinopharm Chemical Reagent Co., Ltd., Shanghai, China) and 50 μL of 2-methyl-3-heptanone (8.16 μg/mL in methanol, Tokyo Chemical Industry Co., Ltd., Tokyo, Japan) as an internal standard. The automatic sampling procedure was as follows: sample was incubated at 45 °C for 10 min to reach equilibrium with shaking at regular intervals (20 s on, 2 s off); 2 cm of DVB/CAR/PDMS SPME fiber was introduced to the headspace environment, followed by the adsorption of volatiles at 45 °C for 40 min; the SPME fiber was inserted into the injection port of a gas chromatography–mass spectrometric (GC–MS) system, followed by the desorption of volatiles at 250 °C for 5 min.

#### 2.3.2. GC–MS Analysis

A GC–MS system (7890 GC & 5975C MS, Agilent Technologies, Santa Clara, CA, USA), fitted with a DBWax (30 m × 0.25 mm i.d. × 0.25 μm, Agilent Technologies) fused silica capillary column, was applied to analyze the HS-SPME isolates basing on our previous work [15]. High-purity helium (99.995%, Qianxi Jingcheng Gas Co., Ltd., Beijing, China), with a constant flow rate of 1.0 mL/min, was used as the carrier gas. The oven temperature was programmed as follows: the initial temperature was set at 40 °C; temperature increased to 60 °C at 2 °C/min; temperature kept at 60 °C for 2 min; temperature rose from 60 °C to 140 °C at 4 °C/min; temperature maintained at 140 °C for 2 min; temperature increased to 250 °C at 10 °C/min; temperature held at 250 °C for 2 min. MS conditions were as follows: ion source, 230 °C; electron energy, 70 eV; scanning mode from m/z 30 to 500.

#### 2.3.3. Identification and Quantitation of the Volatiles

The volatile compounds were identified considering both MS patterns and linear retention indices (LRIs). Mass spectra were compared to those available in the NIST14 libraries. The LRI values were determined and calculated by using n-alkanes (Sigma-Aldrich Chemical Co., Milwaukee, WI, USA) as standards [15]. The compound with match quality ≥80 was regarded as identified if the difference between the calculated and published LRI values was <20. The identified compounds were subsequently quantified by five-point external standard curves [18].

### 2.4. Quantitative Description Analysis (QDA) of Red Raspberry Juice

QDA was performed to further validate the sensory differences in red raspberry juice induced by HHP and HTST sterilization. Seven odor descriptors were selected according to our previous work [9], namely, β-ionone (floral), hexanal (grassy), ethyl butyrate (fruity), furaneol (caramel-like), β-caryophyllene (woody), 4-methylphenol (medicine-like), and butanoic acid (sweaty). The standard reference compound was dissolved in a simulated juice matrix at a concentration of 100 times their odor threshold. A trained panel was required to assess each descriptor by a seven-point linear intensity scale in 0.5 increments from 0 to 3.

### 2.5. Analysis of the Main Enzymes in Fatty Acid Metabolism Pathway from Red Raspberry Juice

The main enzymes related to the fatty acid metabolism pathway, including lipoxygenase (LOX), alcohol dehydrogenase (ADH), hydroperoxide lyase (HPL), alcohol acyltransferase (AAT), were presumed to be involved in the synthesis of C6 aldehyde volatile compounds [19].

#### 2.5.1. Extraction and Assay of LOX

LOX in red raspberry juice was extracted and determined according to the methods of Luo et al. [20], with slight modifications.

Preparation of linoleic acid (LA) working solution: a total of 701.13 mg of LA standard and 1.40 g of Tween 20 were weighed, 5 mL of distilled water was added and mixed evenly, 6 mL of 0.5 M sodium hydroxide solution was added and vortexed until the solution was clear, and then the volume was fixed at 25 mL to obtain an LA standard solution at a concentration of 100 mM. An appropriate volume of LA standard solution was collected and diluted to 5 mM with distilled water to obtain the LA working solution.

Extraction of crude LOX enzyme solution: the LOX extract solution was 50 mM pH 6.8 phosphate-buffered saline (PBS) buffer containing 1.0% polyvinylpolypyrrolidone (PVPP) and 1.0% Triton-100. A total of 0.5 g of each red raspberry juice was weighed, and 5 mL of precooled extract was added and mixed well; the sample was extracted in an ice bath for 30 min and then centrifuged at 16,000× *g* for 20 min at 4 °C, with the supernatant being retained for later use.

Determination of enzyme activity: before the assay, the LA working solution, crude LOX enzyme solution, and boric acid-borax buffer (50 mM, pH 9.0) were incubated at 25 °C for 10 min with shaking. A total of 200 μL of LA working solution, 3 mL of borax-boric acid buffer, and 50 μL of crude enzyme solution were accurately pipetted and mixed by shaking. The absorbance of the samples at 234 nm (A234) was measured at 10 s after the addition of crude enzyme solution. The measurement was performed once every 20 s, and the recording was performed for 2 min. A change of 0.001 units in catalytic absorbance per gram of sample at 25 °C was defined as one LOX enzyme activity unit (U g^−1^ FW).

#### 2.5.2. Extraction and Assay of ADH

ADH in red raspberry juice was extracted and determined based on the method of Ke et al. [21] with slight modifications.

Extraction of crude ADH enzyme solution: the ADH extract solution was 100 mM 2-(N-morpholino) ethanesulfonic acid (MES) buffer (pH 6.5) containing 1.0% PVPP and 2 mM dithiothreitol (DTT). A total of 5 g of each red raspberry juice was weighed, and 15 mL of precooled extract was added and mixed well; the sample was extracted in an ice bath for 30 min and then centrifuged at 16,000× *g* at 4 °C for 20 min, after which the supernatant was retained for later use.

Determination of enzyme activity: 3 mL of MES buffer, 150 μL of 1.6 mM NADH, 150 μL of 80 mM acetaldehyde, and 300 μL of crude enzyme solution were accurately pipetted and mixed by shaking. The absorbance of the samples at 340 nm (A340) was measured at 10 s after the addition of crude enzyme solution. The measurement was performed once every 20 s, and the recording was performed for 2 min. The oxidation of 1 μM NADH per gram of sample at 25 °C was defined as one unit of ADH enzyme activity (U g^−1^ FW).

#### 2.5.3. Extraction and Assay of HPL

HPL in red raspberry juice was extracted and determined basing on the method of Bai et al. [22], with slight modifications.

Extraction of crude HPL enzyme solution: the HPL extract was 150 mM pH 8.0 4-(2-hydroxyethyl)-1-piperazineethanesulfonic acid (HEPES)-KOH buffer containing 250 mM sorbitol, 10 mM EDTA, 10 mM magnesium chloride, 1.0% glycerol, 1.0% PVPP, and 0.1 mM phenylmethylsulfonyl fluoride (PMSF). A total of 3 g of each red raspberry juice was weighed, and 6 mL of precooled HPL extract was added and mixed well; the sample was extracted in an ice bath for 30 min and then centrifuged at 16,000× *g* for 20 min at 4 °C, after which the supernatant was retained for later use.

Preparation of reaction substrate solution: the reaction substrate used in the enzyme activity test was sodium linoleate hydroperoxide. A total of 100 mL of distilled water, 2 mL of 10 mM sodium linoleate, and 0.1 mg/mL LOX enzyme solution (the solvent was 4 mL of 50 mM boric acid-borax buffer (pH 9.0)) were mixed well, and the mixture was placed in a 30 °C water bath for 2 h.

Determination of enzyme activity: 2 mL of 150 mM pH 8.0 HEPES-KOH buffer, 0.75 mL of reaction substrate solution, and 0.15 mL of 1.6 mM NADH were accurately pipetted and incubated at 25 °C with shaking for 10 min, after which 0.1 mL of 0.5 mg/mL ADH enzyme solution and 0.5 mL of crude HPL enzyme solution were added and mixed well by shaking. The absorbance at 340 nm (A340) of the sample was measured at 10 s and 70 s after the addition of crude enzyme solution. A change of 0.001 units in catalytic absorbance per gram of sample at 25 °C was defined as one HPL enzyme activity unit (U g^−1^ FW).

#### 2.5.4. Extraction and Assay of AAT

AAT in red raspberry juice was extracted and determined in accordance with the methods put forward by Ke et al. [21], with slight modifications.

Extraction of crude AAT enzyme solution: the AAT extract solution was 100 mM PBS buffer, pH 7.0, containing 1.0% PVPP. A total of 5 g of each red raspberry juice was weighed, and 10 mL of precooled extract was added and mixed well; the sample was extracted in an ice bath for 30 min and then centrifuged at 16,000× *g* at 4 °C for 20 min, after which the supernatant was retained for later use.

Determination of enzyme activity: 2.25 mL of PBS buffer, 30 μL of 1 M magnesium chloride, 30 μL of 10 mM 5,5′-dithiobis (2-nitrobenzoic acid), 60 μL of 20 mM butanol, and 50 mM acetyl-CoA (60 μL) were accurately pipetted into the solution and incubated at 25 °C for 10 min with shaking. Then, 300 μL of crude enzyme solution was added and mixed by shaking. The absorbance at 412 nm (A412) of the sample was measured at 10 s and 130 s after the addition of crude enzyme solution. A change of 0.001 units in catalytic absorbance per gram of sample at 25 °C was defined as one AAT enzyme activity unit (U g^−1^ FW).

### 2.6. Characterization of the Lipid Profile of Red Raspberry Juice

#### 2.6.1. Analysis of the Fatty Acid Composition

The fatty acids were extracted and determined based on the Chinese Standard (GB/T 5009.168–2016) with slight modifications [23]. The acid hydrolyzed sample was mixed with 10 mL 95% ethanol and then transferred into a separatory funnel containing 50 mL of the mixture of ethyl ether and petroleum. The resulting mixture was shaken for 10 min, followed by a static settlement of 20 min. The fat extract was obtained from the ether layer through rotary evaporation. The fatty acid methyl esters were prepared in accordance with the methods put forward by Wang et al. [24]. The samples were analyzed by a GC–MS system equipped with a DB-17 fused silica capillary column (30 m × 0.25 mm i.d. × 0.25 μm, Agilent Technologies). The oven temperature was programmed as follows: temperature kept at 100 °C for 13 min; temperature increased to 180 °C at 10 °C/min and then held for 6 min; temperature rose to 200 °C at 1 °C/min and then maintained for 20 min; temperature raised to 230 °C at 4 °C/min and then kept for 10.5 min. The fatty acids were identified by means of NIST/Wiley Libraries and quantified based on a five-point external standard calibration curve [20].

#### 2.6.2. Analysis of the Lipid Metabolites

The lipid metabolites of fresh, HHP-, and HTST-treated red raspberry juice were characterized and compared by an untargeted metabolomics strategy as described in our previous work [17]. The pre-frozen juice was freeze-dried in a lyophilizer for 48 h at a temperature of −40 °C ± 2 °C and a vacuum pressure of <5.00 Pa (LGJ-25C, Foring Technology Development Co., Ltd., Beijing, China). The freeze-dried sample was crushed using a mixer mill with a zirconia bead for 1.5 min at 30 Hz (MM 400, Retsch GmbH, Haan, Germany). Afterwards, 100 mg powder was mixed with 1.0 mL 70% aqueous methanol and extracted at 4 °C overnight. Following centrifugation at 10,000× *g* for 10 min, the extracts were absorbed (CNWBOND Carbon-GCB SPE Cartridge, 250 mg, 3 mL; ANPEL Laboratory Technologies Inc., Shanghai, China) and filtrated (SCAA-104, 0.22 μm pore size; ANPEL Laboratory Technologies Inc.). The sample extracts were analyzed using an ultra-performance liquid chromatograph (UPLC)/electrospray ionization (ESI)–MS/MS system (UPLC, Shimadzu CBM30A, Shimadzu Corporation, Kyoto, Japan; MS, AB SCIEX QTRAP 6500, AB SCIEX Instruments, Framingham, MA, USA).

The analytical conditions of UPLC were as follows: column, Waters ACQUITY UPLC HSS T3 C18 (1.8 µm, 2.1 mm × 100 mm); solvent system, ultrapure water containing 0.04% (*v*/*v*) acetic acid as phase A, acetonitrile containing 0.1% (*v*/*v*) acetic acid as phase B; gradient program, time gradient: 0 min → 11 min → 12 min → 15 min, concentration gradient (proportion of phase A): 100% → 5% → 5% → 95%; flow rate, 0.40 mL/min; temperature, 40 °C; injection volume: 2 μL [25].

Linear ion trap (LIT) and triple quadrupole (QqQ) scans were acquired on a triple quadrupole-linear ion trap mass spectrometer (Q TRAP). The ESI source operation parameters were as follows: ion source, turbo spray; source temperature, 500 °C; ion spray voltage (IS), 5500 V; ion source gas I (GSI), gas II (GSII), and curtain gas (CUR) were set at 55, 60, and 25.0 psi, respectively; the collision gas (CAD) was high. Instrument tuning and mass calibration were performed with 10 and 100 μmol/L polypropylene glycol solutions in QqQ and LIT modes, respectively. QqQ scans were acquired as MRM experiments with collision gas (nitrogen) set to 5 psi. The declustering potential (DP) and collision energy (CE) for individual MRM transitions were conducted with further DP and CE optimization. A specific set of MRM transitions was monitored for each period according to the metabolites eluted within this period [26].

Primary and secondary MS data, including the accurate precursor ion (Q1) and production (Q3) values, retention time (Rt), and fragmentation patterns, were subjected to qualitative analysis by comparing them to the data obtained from the self-built database MWDB (MetWare Biological Science and Technology Co., Ltd., Wuhan, China) and publicly available metabolite databases [27].

### 2.7. Statistical Analysis of the Data

SPSS Statistics 22.0 (IBM Corporation, New York, NY, USA) was used to perform a one-way analysis of variance (ANOVA) and Duncan’s multiple range test to determine the significance of the differences among different samples (*p* < 0.05). Raw data obtained from three sample groups were standardized through mean centering and unit variance scaling. Hierarchical clustering analysis (HCA) and orthogonal partial least squares-discriminant analysis (OPLS-DA) were conducted to visualize the metabolic differences among the different sample groups using R software (www.r-project.org/, released on 24 June 2021). The main analytical parameters were as follows: metabolites from different cef files were aligned and assessed by using a retention time window of 0.1% ± 0.15 min and a mass window of 5.0 ppm ± 2.0 mDa; the filter condition was chosen to “retain entities that appeared in at least 65% of samples in at least one condition” in the software; then, parameters of significance analysis and fold change (FC) were adjusted. The *p*-value and fold change were 0.05 and 2.0, respectively. The variable importance in projection (VIP) scores of the OPLS-DA method, with a threshold of 1, were also applied to rank the metabolites that best distinguished between the different sample groups. Among the metabolites with an FC score of ≥2 or ≤0.5, those with a VIP score of ≥1 were considered differential metabolites between the different sample groups. Correlation analysis between differential metabolites, volatile compounds, and main enzymes was conducted through MetaboAnalyst online tools (http://www.metaboanalyst.ca/, accessed on 24 June 2021).

## 3. Results and Discussion

### 3.1. Effect of Sterilization Methods on the C6 Aldehydes Key Aromas and Aroma Profile of Red Raspberry Juice

**Figure 1:**
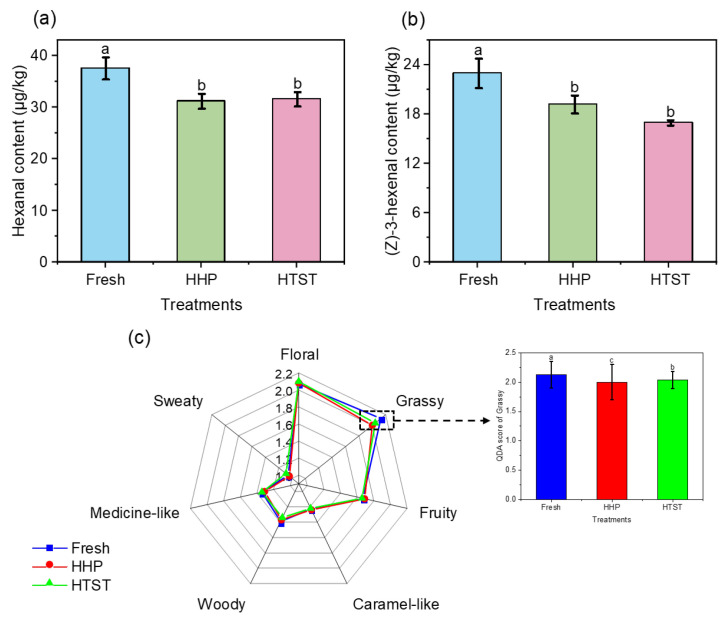
The contents of C6 aldehyde key aroma compounds (**a**,**b**) and aroma profiles (**c**) of red raspberry juices after different sterilization treatments. Different lowercase letters (a–c) denote a significant difference (*p* < 0.05).

The concentrations of all aroma-active compounds identified in variously processed red raspberry juices are detailed in Table S1 of the Supplementary Materials. Specifically, the contents of two key C6 aldehyde compounds are presented separately in Figure 1a,b. Notably, in both HHP and HTST red raspberry juices, the content of hexanal decreased by 16.77% and 13.15%, respectively, while (*Z*)-3-hexenal dropped by 16.48% and 26.13%, respectively. These results indicated that both sterilization methods caused varying degrees of loss in the aromas of C6 aldehydes in red raspberry juice, consistent with our previous findings from studies on pasteurization and HHP sterilization of mango juice [15].

QDA is a powerful method to investigate the effect of aroma compound losses on the overall aroma profile of foods [28]. As illustrated in Figure 1c, most of the sensory attribute scores of fresh, HHP, and HTST red raspberry juices are similar, particularly for the floral, sweaty, medicinal, woody, and fruity notes. However, the grassy odor in HHP and HTST red raspberry juice was significantly lower than that in the fresh sample, aligning with the observed changes in the contents of these two key C6 aldehydes post-sterilization. Therefore, it is speculated that the aroma deterioration of red raspberry juice induced by sterilization is primarily due to the loss of C6 aldehydes.

### 3.2. Effects of Different Sterilization Methods on Key Enzymes in the Fatty Acid Metabolism Pathway of Red Raspberry Juice

**Figure 2:**
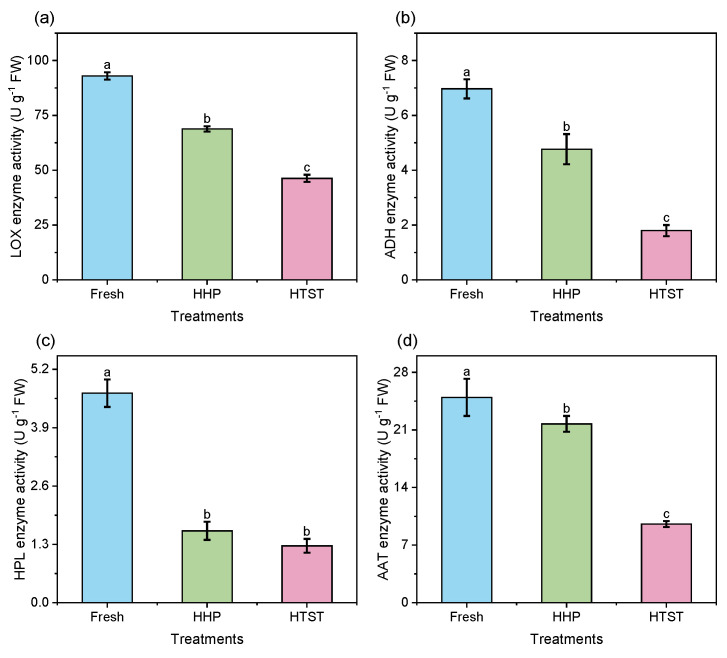
The key enzyme activities of LOX (**a**), ADH (**b**), HPL (**c**), and AAT (**d**) in red raspberry juices after different sterilization treatments. Different lowercase letters (a–c) denote a significant difference (*p* < 0.05).

Aldehyde compounds are primarily derived from the fatty acid metabolism pathway; thus, monitoring and analyzing the activity of key enzymes in this pathway is crucial for understanding how sterilization affects C6 aldehyde compounds. As depicted in Figure 2, the enzymatic activities of LOX, ADH, HPL, and AAT significantly decreased in both HHP and HTST red raspberry juices compared to the control group, suggesting that the reduction in C6 aldehydes may be linked to the diminished enzymatic oxidation of unsaturated fatty acids due to these treatments [29]. Nevertheless, the residual enzyme activities of ADH and AAT in HHP-treated red raspberry juice still reached 68.44% and 87.13%, respectively, indicating that enzymatic conversion of relevant substrates during HHP sterilization is feasible [30]. For instance, (*Z*)-3-hexenal could be converted to (*Z*)-3-hexenol and (*Z*)-3-hexyl acetate by the action of ADH and AAT, potentially explaining the loss of (*Z*)-3-hexenal in HHP-treated juice. Additionally, the residual enzyme activity of LOX in the HTST raspberry juice was 49.78%, with ADH, HPL, and AAT activities all exceeding 25%, showing that HTST has a stronger inactivating effect on these enzymes than HHP. These findings were consistent with the previous studies on melon juice [31] and longan juice [32], which attributed to the reversible nature of enzyme structure damage caused by HHP, allowing partial or complete recovery of their activities during storage.

### 3.3. Effect of Different Sterilization Methods on Aroma Compounds Downstream of the Fatty Acid Metabolism Pathway in Red Raspberry Juice

**Figure 3:**
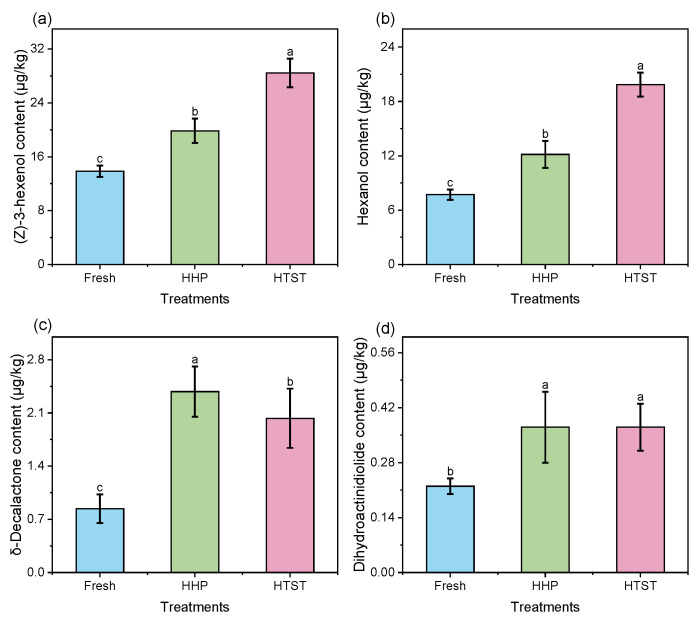
The contents of aroma compounds downstream of the fatty acid metabolism pathway, namely, (Z)-3-hexenol (**a**), hexanol (**b**), δ-decalactone (**c**), and dihydroactinidiolide (**d**), in red raspberry juice after different sterilization treatments. Different lowercase letters (a–c) denote a significant difference (*p* < 0.05).

Among the fatty acid metabolism pathway, substrates such as linoleic and linolenic acids are converted to fatty acid hydroperoxides by LOX and subsequently transformed into aldehydes, alcohols, and esters by HPL, ADH, and AAT [33]. Consequently, analyzing downstream aroma compounds, such as alcohols and lactones, provides insights into the loss pathways of hexanal and (*Z*)-3-hexenal in HHP and HTST sterilization-treated red raspberry juices. As demonstrated in Figure 3a,b, the concentrations of hexanol and (Z)-3-hexenol significantly increased in juices following HHP and HTST treatments. Given that the residual activities of ADH and AAT exceeded 25%, it is speculated that the observed loss of (Z)-3-hexenal and hexanal during processing can be attributed to their enzymatic conversion by ADH and AAT within the LOX pathway [34]. Additionally, Figure 3c,d showed significant increases in δ-decalactone and dihydroactinidiolide contents following HHP and HTST treatments, indicating that conversions related to the β-oxidation pathway occur during sterilization. Considering both the LOX oxidation pathway and the β-oxidation pathway utilize fatty acid peroxides as substrates [35], it is speculated that the loss of C6 and C9 aldehydes may also be related to the competitive inhibition of β-oxidation to LOX oxidation pathway.

### 3.4. Effect of Different Sterilization Methods on the Fatty Acid Composition of Red Raspberry Juice

**Figure 4:**
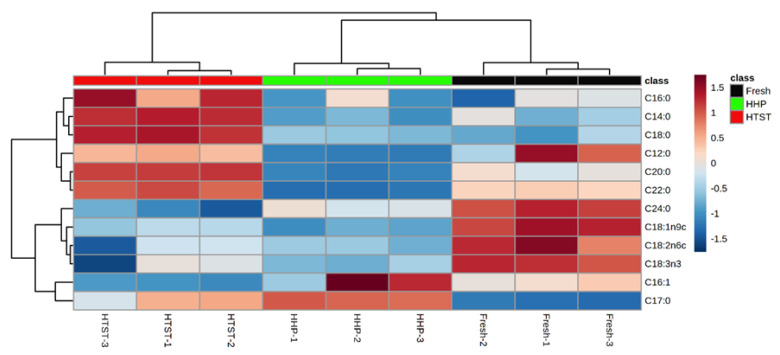
Cluster analysis based on the fatty acid composition of red raspberry juice in red raspberry juices after different sterilization treatments.

To better understand the impact of HHP and HTST sterilization treatments on the fatty acid metabolism pathway, the fatty acid contents in three red raspberry juices were determined. As detailed in Table 1, the juice contains four major unsaturated fatty acids, including linolenic and linoleic acids, as well as eight saturated fatty acids, such as lauric acid. Among these, linoleic acid and linolenic acid have been confirmed to be the precursors of C6/C9 aldehyde aroma compounds in fruits and vegetables, including tomato [36], peach [34], and melon [23]. Post-HHP treatment, the levels of linoleic and linolenic acids decreased significantly, likely due to the enzymatic transformation within the unsaturated fatty acid metabolic pathways, whereas vaccenic acid levels remained unchanged. Conversely, HTST treatment led to a significant reduction in the contents of linoleic, linolenic, and palmitoleic acids, with noticeable increases in vaccenic and palmitic acids. This shift may result from diminished fatty acid dehydrogenase (FAD) activity due to heat exposure, inhibiting the conversion from saturated to unsaturated fatty acids [37]. Cluster analysis of the fatty acid composition in different raspberry juice treatments (Figure 4) revealed fewer significant changes in fatty acids in HHP-treated juice compared to HTST-treated juice, indicating that HHP sterilization better preserves the original fatty acid profile of the raspberry juice than HTST treatment.

### 3.5. Effect of Different Sterilization Methods on Lipid Metabolites in Red Raspberry Juice

**Figure 5:**
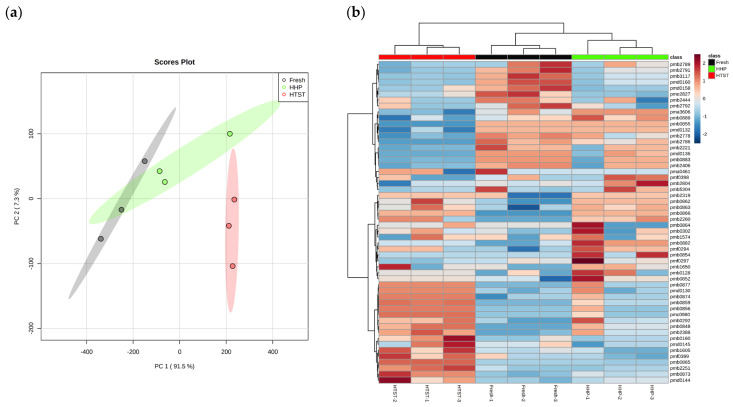
Partial least squares discrimination analysis (**a**) and cluster analysis (**b**) based on the lipid profile of red raspberry juice in raspberry juices after different sterilization treatments.

To clarify the relationship between the C6 aldehydes loss induced by sterilization treatments and the fatty acid metabolic pathway, untargeted metabolomics was employed to analyze the lipid profiles of raspberry juices treated with different sterilization methods, identifying intermediates with significant changes in abundance. A total of 59 lipid metabolites were detected across three red raspberry juice samples, and variations within and between sample groups were examined using PLS-DA analysis (Figure 5a). Remarkably, the first two principal components (PC1 and PC2) explained 98.8% of the overall variance, confirming the efficacy of the PLS-DA model in distinguishing the lipid profiles of the various juices. PC1, which carried significant weight, showed that the Euclidean distance between the HHP-treated juice and the fresh juice was smaller, with partially overlapping confidence ellipses, indicating closer lipid profiles between them. Additionally, cluster analysis was conducted based on the lipid metabolites identified in three red raspberry juice samples, with the results displayed in Figure 5b. The dendrogram at the top showed distinct clustering for juices treated by three different sterilization methods, where the HHP-treated juice exhibited a higher similarity in lipid profiles to the fresh juice. This observation aligns with the PLS-DA findings, suggesting that HHP treatment better preserves the flavor of red raspberry juice compared to HTST treatment.

Table 2 listed 24 differentially metabolized lipids identified after HHP and HTST treatments, with 7 lipids altered in HHP-treated juice (1 upregulated, 6 downregulated) and 22 in HTST-treated juice (9 upregulated, 13 downregulated). This indicated a more pronounced effect of HTST treatment on the lipid profile, aligning with changes observed in the fatty acid composition. Notably, key metabolites, such as 13-peroxy octadecatrienoic acid, 9-peroxy octadecatrienoic acid, 9-hydroxy octadecatriene, and 9,10-epoxy octadecadienoic acid, known to contribute to fruit and vegetable aroma synthesis [38], were notably impacted. These hydroperoxides of fatty acids, typical intermediates in both the LOX and β-oxidation pathways, can transform into C6/C9 aldehydes via the LOX pathway or into hydroxy acids and oxyacids through the β-oxidation pathway, eventually forming lactone aroma compounds [39]. Consequently, significantly downregulated 9-peroxy octadecatrienoic acid may be converted to C6/C9 aldehydes through the LOX pathway or converted to lactone aromas through the β-oxidation pathway, consistent with the increased downstream aroma contents of (*Z*)-3-hexenol, hexanol, δ-decanolactone, and dihydroactinidiolide observed. Moreover, these four intermediates were notably reduced in the HTST-treated juice, indicating that oxidative degradation of unsaturated fatty acids during HTST processing may involve not just thermal oxidation but also enzymatic conversion by key enzymes in the fatty acid metabolic pathway.

### 3.6. Correlation Analysis of Key Aromas of C6 Aldehydes in Red Raspberry Juice and Fatty Acid Metabolic Pathways

**Figure 6:**
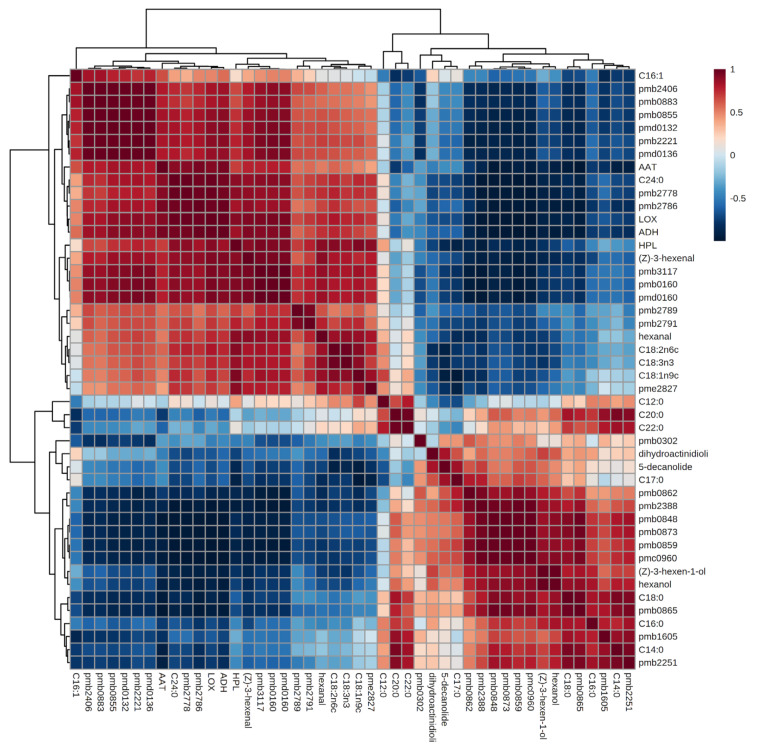
Correlation analysis between C6 aldehyde key aroma compounds and fatty acid metabolism in red raspberry juices after different sterilization treatments.

**Figure 7:**
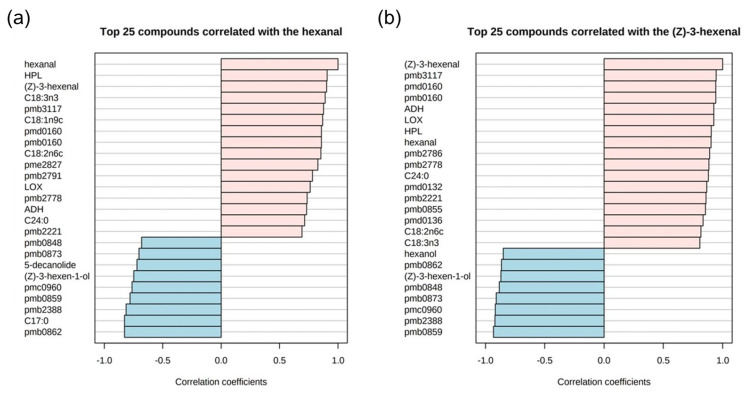
The top 25 correlated metabolites with hexanal (**a**) and (*Z*)-3-hexanal (**b**) in red raspberry juices after different sterilization treatments.

To further elucidate the intrinsic relationship between the loss of C6 aldehydes and fatty acid metabolism pathways during red raspberry juice sterilization, we performed a correlation analysis involving hexanal/(*Z*)-3-hexenal, key enzymes, downstream aroma substances, fatty acid composition, and differentially metabolized lipids. As presented in Figure 6, the changes in the abundance of hexanal and (*Z*)-3-hexenal induced by sterilization treatments were significantly associated with 27 and 34 substances, respectively, with the top 25 correlated substances for each shown in Figure 7a, Figure 7b, respectively.

Noteworthily, changes in hexanal abundance positively correlated with its precursors (linoleic acid and linolenic acid), intermediate products (9-hydroxyperoxy octadecatrienoic acid), and key enzymes (LOX, ADH, HPL) involved in the fatty acid metabolism pathway. Conversely, there was a negative correlation with downstream aroma substances, such as hexanol and (*Z*)-3-hexenol. The synthesis of aroma compounds typically involves the oxidative cleavage of unsaturated and peroxidized fatty acids, leading to the formation of C6/C9 aldehydes [40], which are generally inversely related to their precursor fatty acids—a pattern that diverges from our findings. Considering the negative correlation between hexanal and hexanol (Figure 3b), it is speculated that both the HHP and HTST sterilization treatments may have accelerated the conversion of hexanal, derived from the LOX pathway, to hexanol through ADH dehydrogenation [41]. Alternatively, hexanal also showed a negative correlation with δ-decalactone, a compound typically derived from the β-oxidation pathway of peroxidized fatty acids, suggesting that the competitive inhibition of β-oxidation for LOX conversion could also contribute to the loss of hexanal in sterilized red raspberry juices.

Similarly, changes in (Z)-3-hexenal abundance after sterilization were significantly correlated with acid precursors (linoleic acid and linolenic acid), intermediates (9-hydroxy octadecatrienoic acid and 9,10-epoxy octadecadienoic acid), and key enzymes (LOX, ADH, HPL), while significantly negatively correlated with downstream aroma substances, specifically (Z)-3-hexenol. These findings align with the studies reported by Pei et al. [23,42], which also demonstrated that the C6 and C9 aldehydes identified in HHP-treated melon juices exhibited stronger correlations with fatty acids and aromatic compounds downstream of the fatty acid metabolic pathway. Within the LOX pathway, (*Z*)-3-hexenal can be converted to (E)-2-hexenal and hexenal via cracking by HPL or to (*Z*)-3-hexenol via dehydrogenation by ADH. Consequently, the loss of (*Z*)-3-hexenal is predominantly due to its conversion and decomposition within the LOX pathway, and the decomposition degree of (*Z*)-3-hexenal is greater than that of hexenal. In addition, the content of (*Z*)-3-hexenal in HTST-treated raspberry juice was significantly lower than in HHP-treated juice, indicating more substantial (*Z*)-3-hexenal loss during HTST processing. This discrepancy may stem from the further reduced fatty acid dehydrogenase (FAD) activity during HTST treatment, which hinders the enzymatic oxidation of saturated fatty acids [43], subsequently reducing the availability of unsaturated fatty acid precursors for LOX pathway conversion and resulting in a formation rate of (*Z*)-3-hexenal much lower than its decomposition rate.

## 4. Conclusions

Building on our previous characterization of the flavor profile of raspberry juice, this study comparatively analyzed the effects of HHP and HTST sterilization treatments on the key aromas of C6 aldehydes in raspberry juice. The regulatory mechanisms were explored through nontargeted metabolomics analysis of fatty acids. Specifically, the external standard method showed that the HHP and HTST treatments could cause a significant reduction in the hexanal and (*Z*)-3-hexenal contents in raspberry juice, and QDA revealed that this loss caused sensory differences in the grassy aroma attribute. The key enzyme activities, downstream aroma, fatty acid composition, and lipid metabolites in the fatty acid metabolic pathway in different raspberry juices were further characterized. These results revealed that the C6 aldehydes loss was closely related to the dynamic conversion of the fatty acid metabolic pathway. Although oxidatively degraded unsaturated fatty acids are partially converted to C6 aldehydes, competitive inhibition from β-oxidation and impeded enzymatic oxidation of saturated fatty acids lead to a formation rate that is lower than the decomposition rate, ultimately resulting in the loss of hexanal and (*Z*)-3-hexenal, the two key C6 aldehydes, during processing.

## Figures and Tables

**Table 1:** Fatty acid contents (mg/kg) of red raspberry juices after different sterilization treatments. Values with different lowercase letters in the same row denote a significant difference (*p* < 0.05).

Chemical Name	Structure Simple Formula	Alternative Name	Saturability	Fresh	HHP	HTST
Dodecanoic acid	C12:0	Lauric acid	Saturation	2.85 ± 0.07 b	1.53 ± 0.04 a	2.69 ± 0.05 b
Tetradeconic acid	C14:0	Myristic acid	Saturation	2.61 ± 0.19 c	2.38 ± 0.09 a	3.46 ± 0.03 b
Hexadecanoic acid	C16:0	Palmitic acid	Saturation	68.13 ± 0.77 b	68.01 ± 0.64 b	69.78 ± 0.52 a
(*Z*)-9-hexadecenoic acid	C16:1	Palmitoleic acid	Monounsaturated	2.45 ± 0.04 b	2.62 ± 0.28 b	2.19 ± 0.01 a
Heptadecanoic acid	C17:0	Perlitic acid	Saturation	2.03 ± 0.01 b	2.41 ± 0.01 b	2.30 ± 0.07 a
Octadecanoic acid	C18:0	Vaccenic acid	Saturation	12.30 ± 0.52 b	12.50 ± 0.12 b	15.70 ± 0.16 a
(*Z*)-9-octadecenoic acid	C18:1n9c	Oleic acid	Monounsaturated	42.79 ± 1.14 c	29.01 ± 0.84 a	31.97 ± 0.88 b
(*Z, Z*)-9, 12-octadecadienoic acid	C18:2n6c	Linoleic acid	Polyunsaturated	58.12 ± 0.76 b	54.76 ± 0.24 a	54.73 ± 1.31 a
Eicosanoic acid	C20:0	Arachidic acid	Saturation	4.58 b ± 0.05 c	4.17 ± 0.02 b	5.02 ± 0.01 a
(*Z, Z, Z*)-9, 12, 15-octadecatrienoic acid	C18:3n3	Linolenic acid	Polyunsaturated	59.95 ± 0.65 b	51.79 ± 0.76 a	52.12 ± 4.03 a
Docosanoic acid	C22:0	Behenic acid	Saturation	3.81 ± 0.04 c	2.26 ± 0.04 b	4.58 ± 0.09 a
Tetracosanoic acid	C24:0	Taric acid	Saturation	2.99 ± 0.03 c	2.73 ± 0.03 b	2.51 ± 0.07 a

**Table 2:** The metabolized lipids identified in red raspberry juices after different sterilization treatments.

Retention Time (min)	Precursor Ions (Da)	Product Ions (Da)	Index	Chemical Name	Ionization Model	HHP	HTST				
						VIP	FC	Variation Trend	VIP	FC	Variation Trend
3.69	126.10	96.00	pmb0302	2-aminoethyl phosphate	[M + H]+	1.30	2.21	up			
6.77	440.10	184.50	pmb0862	Lysophosphatidyl choline 12:1	[M + H]+				1.32	2.07	up
6.77	239.00	223.00	pme2827	Palmitaldehyde	[M − H]−	1.53	0.36	down	1.14	0.48	down
6.95	318.30	60.10	pmb2221	4-hydroxysphingosine	[M + H]+				1.34	0.31	down
7.54	309.00	209.00	pmb2789	13-Peroxyoctadecatrienoic acid	[M − H]−				1.04	0.40	down
7.55	309.10	209.10	pmb2791	9-Hydroxyperoxyoctadecatrienoic acid	[M − H]−	1.30	0.50	down	1.07	0.45	down
7.60	353.30	261.10	pmb0160	Monoacylglyceride (acyl 18:3) isomer 5	[M + H]+	1.24	0.44	down	1.04	8.23	up
7.60	694.10	353.70	pmb2251	Monoacylglyceride disaccharide (18:1)	[M + H]+	1.14	0.00	down			
7.71	518.20	184.10	pmb0865	Lysophosphatidylcholine 18:3 (2nisomerism)	[M + H]+				1.34	2.94	up
7.88	494.30	184.20	pmb0848	Lysophosphatidylcholine 16:1 (2nisomerism)	[M + H]+				1.33	2.20	up
8.25	520.00	184.50	pmb0873	Lysophosphatidylcholine 18:2 (2nisomerism)	[M + H]+				1.32	2.92	up
8.42	293.00	275.00	pmb2786	9-Hydroxyoctadecatrienoic acid	[M − H]−				1.34	0.01	down
8.45	353.30	261.40	pmb1605	Monoacylglyceride (acyl 18:3) isomer 3	[M + H]+	1.14	0.35	down	1.25	2.71	up
8.73	452.30	255.20	pmd0160	Lysophosphatidyl ethanolamine 16:0 (2n isomerism)	[M − H]−				1.34	0.18	down
8.74	452.00	255.30	pmb3117	Lysophosphatidyl ethanolamine 16:0	[M − H]−	1.26	0.39	down	1.34	0.14	down
8.80	496.30	184.20	pmb0855	Lysophosphatidylcholine 16:0	[M + H]+				1.33	0.45	down
8.80	496.30	184.00	pmd0132	Lysophosphatidylcholine 16:0 (2n isomerism)	[M + H]+				1.33	0.45	down
8.91	522.40	184.00	pmb0859	Lysophosphatidylcholine 18:1 (2n isomerism)	[M + H]+				1.34	2.04	up
8.91	524.10	184.00	pmb2388	Lysophosphatidylcholine 18:0 (2n isomerism)	[M + H]+				1.34	2.19	up
8.91	544.40	485.20	pmc0960	Lysophosphatidylcholine 20:4	[M + H]+				1.34	2.16	up
8.96	295.00	277.30	pmb2778	9,10-Epoxy octadecadienoic acid	[M − H]−				1.33	0.37	down
9.34	510.10	184.40	pmb2406	Lysophosphatidylcholine 17:0	[M + H]+				1.33	0.27	down
9.86	482.10	341.70	pmb0883	Lysophosphatidyl ethanolamine 18:0	[M + H]+				1.33	0.17	down
9.91	524.40	184.00	pmd0136	Lysophosphatidylcholine 18:0	[M + H]+				1.34	0.17	down

## Data Availability

The data presented in this study are available on request from the corresponding author. The data are not publicly available due to ongoing research using a part of the data.

## Supplementary Materials

The following supporting information can be downloaded at https://www.mdpi.com/article/10.3390/foods13101574/s1, Table S1: The 12 aroma-active compounds in red raspberry juices after different sterilization treatments.
