# Supplementary material for: Decoding the Effects of High Hydrostatic Pressure and High-Temperature Short-Time Sterilization on the Volatile Aroma Profile of Red Raspberry Juice
Source: Foods. 2024 May 18;13(10):1574. doi: 10.3390/foods13101574 (PMC11121533; doi:10.3390/foods13101574)
Supplement: Supplementary file 1 [file foods-13-01574-s001.zip › foods-3004744-supplementary.pdf]

Supplementary materials

Table S1 The 12 aroma-active compounds in red raspberry juices after different sterilization treatments

no.	compound [a]	CAS Registry No.	LRI [b]		odor description [c]	I methods [d]	concentration (μg/kg)		
			DB-5	DB-WAX			Fresh	HHP	HTST
1	hexanal	66-25-1	-	--	grass, cucumber, fat	MS, O, S	37.55 ± 2.14	31.21 ± 1.46	31.57 ± 1.38
2	(*Z*)-3-hexenal	6789-80-6	804	1148	grass, leaf, sweat	MS, LRI, O, S	22.98 ± 1.79	19.21 ± 1.08	16.99 ± 0.33
3	eucalyptol	470-82-6	1033	1211	rosin, mint, camphor	MS, LRI, O, S	10.15 ± 1.21	10.87 ± 0.97	10.06 ± 1.10
4	(*E*)-2-hexenal	6728-26-3	856	1217	leaf, vegetable, earth	MS, LRI, O, S	179.85 ± 7.53	235.57 ± 11.53	291.77 ± 14.98
5	(*Z*)-3-hexen-1-ol	928-96-1	-	1385	grass, sweat	MS, LRI, O, S	2.03 ± 0.13	2.20 ± 0.19	2.01 ± 0.05
6	1-octen-3-ol	3391-86-4	978	1453	soap, mushroom, milk	MS, LRI, O, S	2.09 ± 0.07	2.02 ± 0.24	1.57 ± 0.12
7	theaspirane	36431-72-8	1296	1533	tea, peel	MS, LRI, O, S	3.05 ± 0.17	4.63 ± 0.36	3.88 ± 0.26
8	linalool	78-70-6	-	1550	passion fruit, flower	MS, LRI, O, S	17.09 ± 1.11	15.14 ± 2.45	8.92 ± 1.20
9	damascenone	23696-85-7		1818	sweet, rose, honey	MS, LRI, O, S	5.27 ± 0.70	8.92 ± 1.51	10.22 ± 0.52
10	dihydro-β-ionone	17283-81-7	1431	1836	sweet, violet, wood	MS, LRI, O, S	1.47 ± 0.24	1.74 ± 0.25	1.81 ± 0.22
11	α-ionone	127-41-3	1426	1851	flower, violet, wood	MS, LRI, O, S	11.49 ± 1.52	8.73 ± 1.63	5.65 ± 0.7
12	β-ionone	79-77-6	1483	1937	violet, flower, raspberry	MS, LRI, O, S	87.2 ± 4.43	66.55 ± 4.65	85.82 ± 7.43

[a] The 12 volatile compounds were identified as major aroma-active compound in our previous work[9].

[b] Retention indices on DB-5 and DB-Wax columns were determined as described[9].

[c] Odor description perceived by the judges during GC-O-MS analysis.

[d] MS, identified by MS spectra; LRI, identified by comparison of their LRI on two columns (DB-5 and DB-Wax) with published data; O, identified by comparison of their odor description with the authentic compounds via GC–O-MS; S, identified by comparison to standards.
